# Use of a new off-the-shelf 3D-printed trabecular titanium acetabular cup in Chinese patients undergoing hip revision surgery: Short- to mid-term clinical and radiological outcomes

**DOI:** 10.1186/s12891-022-05596-z

**Published:** 2022-07-04

**Authors:** Guangqian Shang, Shuai Xiang, Cuicui Guo, Jianjun Guo, Peng Wang, Yingzhen Wang, Hao Xu

**Affiliations:** Department of Joint Surgery, The Affiliated Hospital of Qingdao University, No. 59, Haier Road, Qingdao, 266000 Shandong China

**Keywords:** Reoperation, Hip, Trabecular titanium acetabular cup, 3D printing

## Abstract

**Background:**

Revision total hip arthroplasty (THA) has been a challenge for surgeons. The purpose of this study was to explore the short-to mid-term clinical and radiological outcomes of Chinese patients who underwent revision THA using a new off-the-shelf three-dimensional (3D)-printed trabecular titanium (TT) acetabular cup by comparison with a conventional porous coated titanium acetabular cup, to provide a reference for the recommendation of this prostheses.

**Methods:**

A retrospective analysis of 57 patients (57 hips) who received revision THA was performed from January 2016 to June 2019. A total of 23 patients received 3D-printed cups (observation group) and 34 patients received non-3D-printed cups (control group). Clinical scores including Visual Analogue Scale (VAS), Harris Hip Score (HHS) and Short Form 36 (SF-36), upward movement of the hip center of rotation(HCOR)and limb-length discrepancy (LLD), stabilization and bone ingrowth of cups were compared between two groups. The multivariate linear regression was used to determine the factors potentially influencing the HHS score. Postoperative complications in the two groups were also recorded.

**Results:**

All 57 patients were routinely followed up. The average follow-up durations in the control and observation groups were 43.57 ± 13.68 (24–65) months and 41.82 ± 11.44 (24–64) months, respectively (*p* = 0.618). The postoperative clinical scores significantly improved in both groups compared to the preoperative scores (*p* < 0.001). The VAS score did not significantly differ between the groups at 3 (*p* = 0.946) or 12 (*p* = 0.681) months postoperatively, or at the last follow-up (*p* = 0.885). The HHS score did not significantly differ between the groups at 3 months (*p* = 0.378) postoperatively but differed at 12 months (*p* < 0.001) postoperatively and the last follow-up (*p* < 0.001). The SF-36 score did not significantly differ between the groups at 3 months (*p* = 0.289) postoperatively, but was significantly different at 12 months (*p* < 0.001) postoperatively and the last follow-up (*p* < 0.001). Compared with the control group, the postoperative recovery of HCOR and LLD was better in the observation group. All cups remained stable, with no loosening throughout the follow-up period. But the observation group had a significantly better rate of bone ingrowth compared to the control group (*p* = 0.037). Multivariate linear regression analysis showed that different cup types, upward movement of the HCOR, and LLD influenced the HHS score at the last follow-up (*p* < 0.001, *p* = 0.005, respectively). None of the patients exhibited severe postoperative complications.

**Conclusion:**

The new off-the-shelf 3D-printed TT acetabular cup demonstrated encouraging short-to mid-term clinical outcomes in Chinese patients. It can effectively relieve pain, improve hip function, provide satisfactory biological fixation and high survival rate. But further follow up is necessary to assess its long-term outcomes.

## Background

Total hip arthroplasty (THA) is an effective surgery for end-stage hip diseases, such as avascular necrosis of femoral heads and osteoarthritis, because it relieves pain and improves joint function and quality of life [1]. Although the longevity of prostheses has significantly improved because of advancements in surgical techniques and prosthesis design, some protheses need to be replaced over time [2]. Because of the aging population and primary THA being performed in younger patients, the number of revision surgeries is gradually increasing. It is estimated that the number of revision THAs performed in the USA will double by 2026 [3].

The most common indication for revision THA is aseptic loosening (AL) of acetabular cups [4]. One of the major objectives of revision surgery is early and long-term stable fixation of implants. Acetabular bone defects are also a challenge for surgeons, even the most experienced ones. These defects can affect primary mechanical stability and cause secondary bone ingrowth, which may necessitate re-revision surgery. The Paprosky classification, based on the integrity of Kohler’s line, teardrop or ischium osteolysis, and acetabular component migration, is the most commonly used classification system [5]. A study of 1,094 cases of revision THA reported severe acetabular bone defects in 17% of cases, but the surgical failure rate was as high as 30% [6].

In recent years, three-dimensional (3D) printing technology has been widely used in medical fields, especially orthopedics [7]. Traditionally, metal implants were created by “formative shaping” or “subtractive manufacturing”; however, 3D printing technology has made additive manufacturing a reality. Electron beam melting (EBM), an important 3D printing technology, allows titanium to be melted at nearly 2,000℃ and produces acetabular cups with a specific design and controlled porous surface. These cups reduce the elastic modulus and have biological characteristics similar to those of subchondral bone [8, 9].

In this study of Chinese patients who underwent revision surgery, we compared a new off-the-shelf 3D-printed trabecular titanium (TT) cup to a conventional titanium cup with a porous coated back.

## Methods

### Inclusion and exclusion criteria

After the study had been approved by our Institutional Review Board (QYFYWZLL-26263), we retrospectively collected data from 57 patients (57 hips) who underwent revision THA between January 2016 and June 2019 at the Affiliated Hospital of Qingdao University (Qingdao, China). We included patients who were diagnosed with AL or periprosthetic joint infection (PJI) according to Musculoskeletal Infection Society criteria after unilateral primary THA [10]. We excluded patients with insufficient neuromuscular function, including cerebral palsy, poliomyelitis, Parkinson’s disease, Charcot joint, cerebrovascular accidents, spinal injury, diastrophic dysplasia and myelomeningocele, as this may cause postoperative hip instability or gait abnormality, periprosthetic fracture after primary THA due to trauma, uncontrolled PJI (Between first stage and second stage, patients presented with symptoms of active infection or no decreasing trend in ESR and CRP levels after 2 weeks of the antibiotic holiday), or infections other than PJI.

### Acetabular implants

**Fig. 1:**
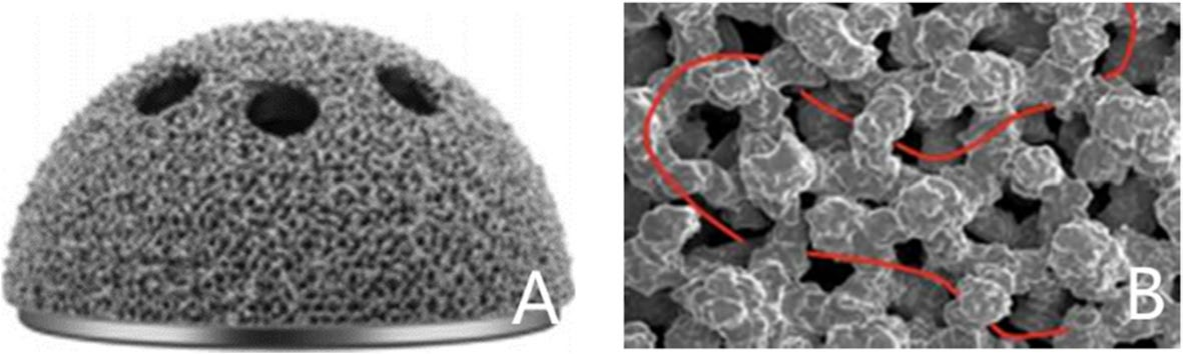
The pictures showed the appearance of three-dimensional printed trabecular titanium acetabular cup (**A**) and the SEM image of its interconnected trabecular titanium cellular solid structure (**B**). SEM: scanning electron microscope

Based on the acetabular revision material used, patients were divided into two groups. Patients who received a 3D-printed TT acetabular cup (Aikang Corp., Beijing, China) were included in the observation group (Fig. 1). Because of the continuity between the metal solid and surface porous layers, it provided greater resistance against detachment and corrosion. The implant had a 1.5-mm-thick trabecular-like porous titanium construct on the back surface, with an average porosity of 80% and pore size of 600–1,000 μm. Patients who received a conventional porous titanium-coated acetabular cup (Reflection Acetabular System, Smith and Nephew, Memphis, TN, USA), were included in the control group. This implant had a porous coating of sintered titanium beads with a mean porosity of 40% and pore size of 250 μm.

### Surgical procedures

Patients received general anesthesia in combination with fascial iliac block. All revision surgeries were performed by three senior surgeons, using a modified Hardinge approach. After exposure, the original prostheses or spacers, periacetabular scar tissues, cement, and particles were thoroughly removed. Stems can be retained if prostheses were stable or revised if loosening was observed on preoperative imaging studies or intraoperatively. The degree of acetabular bone defect was assessed by direct observation. The acetabular rotation center was identified, and the acetabulum was sequentially reamed using dedicated hemispherical reamers until blood oozed evenly from the bone surface. The bone defect was reassessed in terms of the need for metal augment or structural bone graft to fill the cavitary defects, and trial implants were inserted to assess coverage, impingement, and stability. In cases of severe acetabular bone defects, metal augment or structural bone graft from autologous iliac bone was used to fill the defect between the cup’s dome and host bone, followed by screws to ensure stability of augment or bone graft. According to the preoperative plan, the definitive cup was inserted at the appropriate abduction (40 ± 10°) and anteversion (15 ± 5°) angles. The cup was stabilized by 1–3 screws inserted through the holes.

### Postoperative recovery

Multimodal analgesia was administered to the patients according to clinical guidelines. The analgesia included intravenous flurbiprofen axetil and oral oxycodone. Cephalosporins were administered for 48 h postoperatively to prevent infections; patients who were allergic to cephalosporin were administered clindamycin. Rivaroxaban (25 mg/day) was given orally within 12–24 h after the operation, and continued for a month to reduce the risk of deep venous thrombosis. Functional exercises were gradually introduced over time. Patients were made partial weight-bearing until 6 weeks, and full weight-bearing was allowed thereafter.

### Clinical and radiographic assessment

Visual analog scale (VAS), Harris Hip Score (HHS), and Short-Form 36 (SF-36) values were recorded preoperatively, 3 and 12 months postoperatively, and at the last follow-up for patients in both groups. These assessments were performed by the same clinical staff, who did not participate in the surgery.

Anteroposterior hip joint radiographs were obtained to evaluate upward movement of the hip center of rotation (HCOR) and limb-length discrepancy (LLD) preoperatively, 1 day postoperatively, and at the last follow-up. Bilateral teardrop connection was used as the reference line for these assessments; the upward movement distance of HCOR was defined as the vertical distance between HCOR on the affected side and the reference line, and LLD was defined as the difference in vertical distance between the vertices of bilateral lesser trochanters and the reference line.

**Fig. 2:**
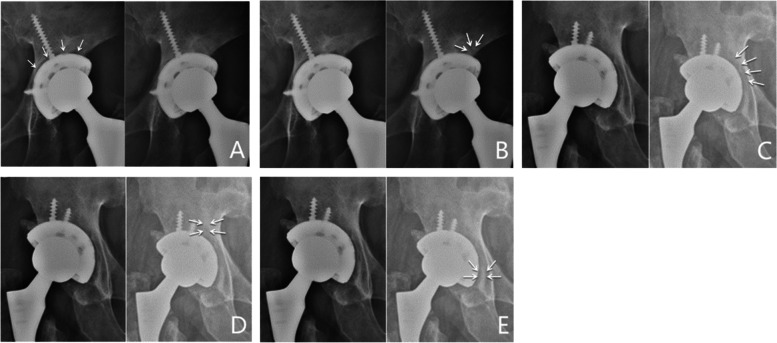
Representative cases with radiographic signs of osseointegration as defined by the Anderson Orthopedic Research Institute, inlcluding absence of RLL **A** presence of superolateral buttresses **B** presence of medial stress-shielding **C** presence of radial trabeculae **D** and presence of inferomedial buttresses **E**. RLL: radiolucent line

Using the zonal analysis of DeLee and Charnley [11], cup position was evaluated according to the width of the radiolucent line (RLL) and changes in abduction angle and displacement distance. Cup stabilization was defined as the width of a RLL less than 1 mm in two zones, no RLL in at least two zones, and no displacement. Cup loosening was defined as a change in abduction angle of more than 10° or cup migration of more than 6 mm in any direction. Bone ingrowth of cups was evaluated according to the criteria defined by the Anderson Orthopedic Research Institute [12]. Osseointegration was diagnosed if at least three of the following signs were observed: absence of RLLs; presence of superolateral buttresses; presence of medial stress-shielding; presence of radial trabeculae; and presence of inferomedial buttresses (Figs. 2).

Postoperative complications in the two groups were also recorded.

### Statistical analyses

Statistical analyses were performed using SPSS software (version 24.0; IBM Corp., Armonk, NY, USA). The Shapiro–Wilk test was used to determine the normality of the data. Continuous variables are expressed as means and standard deviations (SDs). Student’s t-test was used to compare continuous variables between the two groups. Clinical scores, as well as upward movement of the HCOR and LLD, were compared between groups using analysis of variance. Multiple comparisons were made using the least significant difference (LSD) test. Categorical variables are presented as frequencies and constituent ratios, and were compared using the χ^2^ test. Multivariate linear regression was used to determine the factors that potentially influenced the HHS score. *P* < 0.05 indicated statistical significance.

## Results

### Demographic characteristics

Twenty-three patients received 3D-printed cups (observation group) and 34 received non-3D-printed cups (control group). No patients were lost to follow up, and 57 patients were routinely followed up either in the outpatient department or over the telephone. The average follow-up durations in the control and observation groups were 43.57 ± 13.68 (24–65) months and 41.82 ± 11.44 (24–64) months, respectively. The acetabular defects were graded according to the Paprosky classification: 4 were Paprosky I, 9 Paprosky IIa, 5 Paprosky IIb, 1 Paprosky IIc, and 4 Paprosky IIIa in the observation group, whereas 6 were Paprosky I, 13 Paprosky IIa, 8 Paprosky IIb, 2 Paprosky IIc, and 5 Paprosky IIIa in the control group. To repair the bone defect, four patients in the observation group required augment graft, one required structural bone graft from the ilium, and five required combined augment and structural bone grafts. In the control group, six patients required augment graft, three required structural bone graft, and six required combined augment and structural bone grafts.

**Fig. 3:**
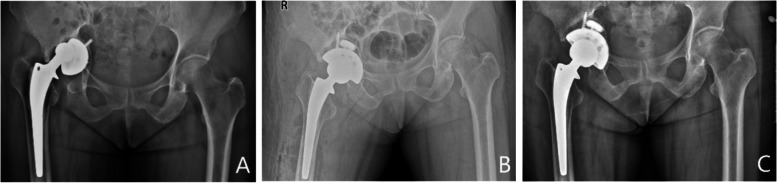
Fourteen years after primary total hip arthroplasty, a 69-year-old woman underwent revision surgery using a three-dimensional printed trabecular titanium acetabular cup combined with metal augment and structural bone due to aseptic loosening. **A** Pre-operative radiograph showing a large acetabular defect. **B** Radiograph obtained at 1 day postoperatively showed that the appropriate hip center of rotation had been restored. **C** Radiograph obtained at 30 months postoperatively showed that the cup and augment were stable, and the bone graft was incorporated

**Fig. 4:**
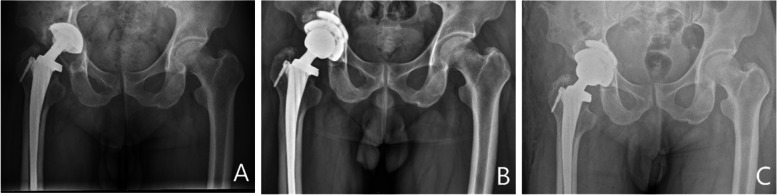
Five years after primary total hip arthroplasty, a 47-year-old man underwent revision surgery using a three-dimensional printed trabecular titanium acetabular cup and augment due to aseptic loosening. **A** Pre-operative radiograph showing a large acetabular defec. **B** Radiograph obtained at 1 day postoperatively. **C** Radiograph obtained at 25 months postoperatively showing the absence of a radiolucent line

As shown in Table 1, no significant differences were observed in age, body mass index (BMI), gender, laterality, mean follow-up duration, reason for revision surgery, or Paprosky classification between the two groups. Figures 3 and 4 present representative images of 3D-printed TT acetabular cups.

**Table 1:** Demographic data of patients (M ± SDs)

	Observation group (*n* = 23)	Control group (*n* = 34)	*P* value
Age (year)	70.35 ± 8.10	71.62 ± 10.23	0.604
BMI (Kg/ m^2^)	25.61 ± 2.80	26.26 ± 2.49	0.360
Gender (number, %)			0.791
Male	10, 43.48	14, 41.18	
Female	13, 56.52	20, 58.82	
Laterality (number, %)			0.550
Left hip	11, 47.83	15, 44.12	
Right hip	12, 52.17	19, 55.88	
Mean follow-up time (month)	43.57 ± 13.68	41.82 ± 11.44	0.605
Reason for revision (number, %)			0.684
Aseptic loosening	17, 73.91	24, 70.59	
Infection	6, 26.09	10, 29.41	
Paprosky classification (number, %)			0.786
Type I	4, 17.39	6, 17.65	
Type II (A + B + C)	15, 65.22	23, 67.65	
Type III (A + B)	4, 17.39	5, 14.70	

*M* ± *SDs* Mean ± standard deviations, *BMI* Body mass index

### Clinical scores

**Fig. 5:**
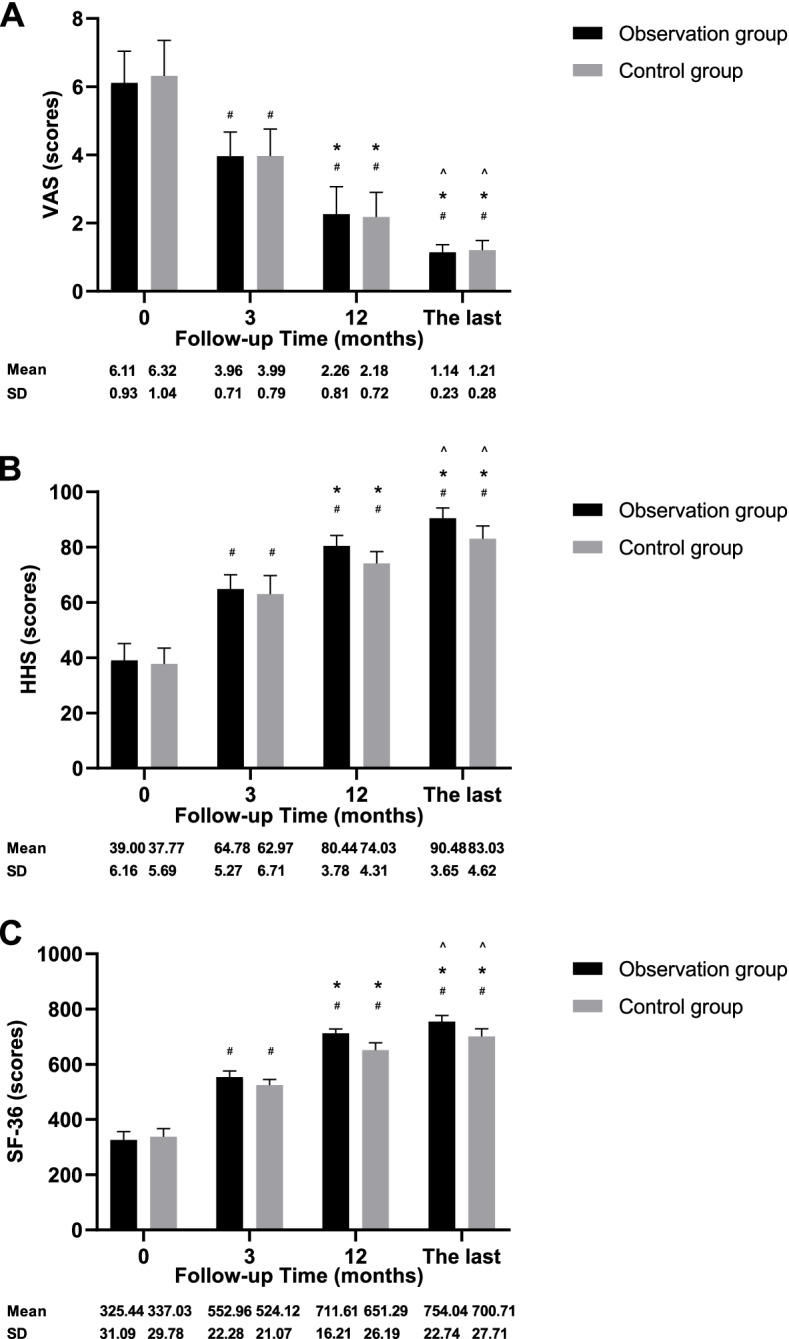
The graphs showed the changes of the VAS (**A**), HHS (**B**) and SF-36 (**C**) scores preoperatively, 3 and 12 months postoperatively, and at the last follow-up for patients in both groups. # p < 0.05 vs before surgery; * p < 0.05 vs 3 months after surgery; ^ p < 0.05 vs 12 months after surgery. VAS: Visual Analogue Scale, HHS: Harris Hip Score, SF-36: Short Form 36

Figure 5 depicts the clinical scores for both groups. The postoperative VAS, HHS, and SF-36 scores significantly improved in both groups compared to the preoperative scores (*p* < 0.001). The VAS score did not significantly differ between the groups at 3 (*p* = 0.946) or 12 (*p* = 0.681) months postoperatively, or at the last follow-up (*p* = 0.885). The HHS score did not significantly differ between the groups at 3 months (*p* = 0.378) postoperatively, but was significantly different at 12 months (*p* < 0.001) postoperatively and the last follow-up (*p* < 0.001). The SF-36 score did not significantly differ between the groups at 3 months (*p* = 0.289) postoperatively, but was significantly different at 12 months (*p* < 0.001) postoperatively and the last follow-up (*p* < 0.001).

### HCOR and LLD

**Fig. 6:**
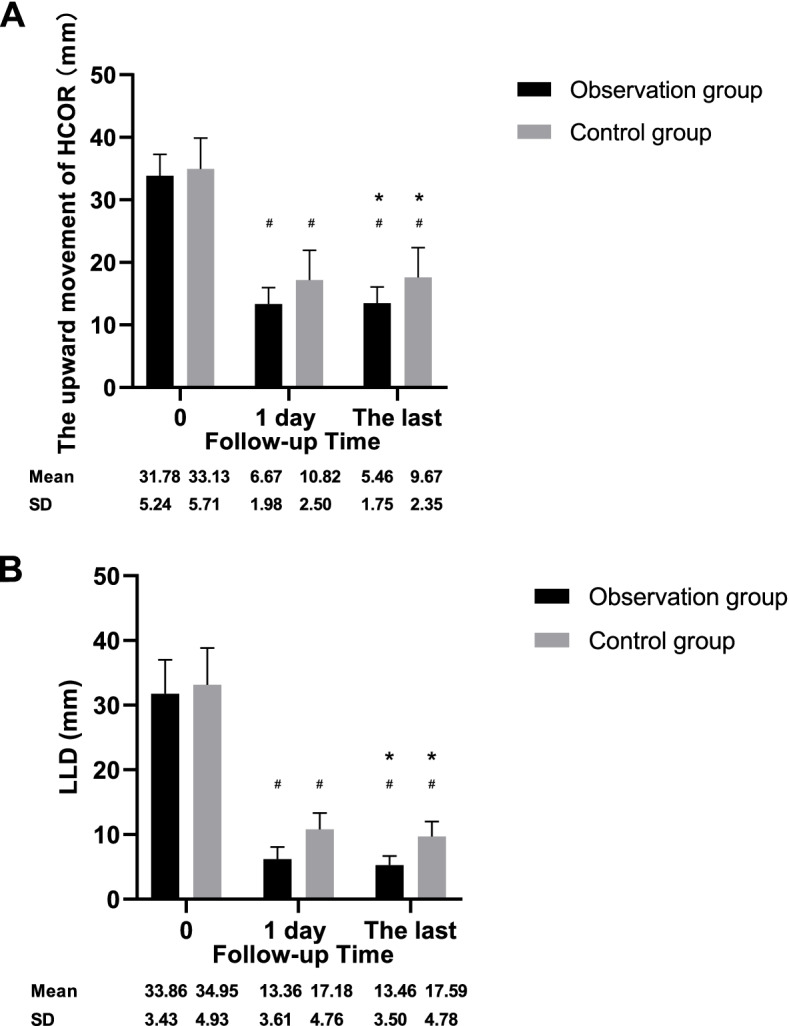
The graphs showed the changes of upward movement distance of the HCOR (**A**) and LLD (**B**) preoperatively, 1 day postoperatively, and at the last follow-up for patients in both groups. # p < 0.05 vs before surgery; * p < 0.05 vs 1 day after surgery. HCOR: hip center of rotation, LLD: limb-length discrepancy

Figure 6 depicts the upward movement of the HCOR and LLD in both groups. In both groups, these values were significantly lower at 1 day postoperatively and the last follow-up compared to the preoperative values (*p* < 0.001). However, postoperative recovery was better in the observation group (*p* < 0.001).

### Stabilization and bone ingrowth of cups

All cups remained stable, with no loosening throughout the follow-up period. The cup abduction angle did not change between 1 day postoperatively and the last follow-up in the observation (*p* = 0.888) or control (*p* = 0.963) group (Table 2). Nonprogressive RLLS appeared in the patients during the follow-up period.

**Table 2:** The cup abduction angle between the observation and control groups (M ± SDs)

	1 day postoperatively	the last follow-up	*P* value
Observation group	39.35 ± 5.41	39.12 ± 5.40	0.888
Control group	40.07 ± 5.51	40.14 ± 5.40	0.963
*P* value	0.625	0.490	—

*M* ± *SDs* Mean ± standard deviation

Based on the bone ingrowth criteria, 2 cups in the observation group had two signs, 17 had three signs, and 4 had four signs preoperatively. However, postoperatively, 10 cups had two signs, 22 had three signs, and 2 had four signs at the last follow-up. The observation group had a significantly better rate of bone ingrowth compared to the control group (91.30 and 70.59%, respectively; *p* = 0.037) (Table 3).

**Table 3:** Bone ingrowth between the observation and control groups at the last follow-up

	bone ingrowth criteria		Total	Rate of bone ingrowth
	< 3 signs	≥ 3 signs		
Observation group	2	21	23	91.30%
Control group	10	24	34	70.59%
Total	12	45	57	78.95%

At least 3 signs can be considered as good bone ingrowth. The χ^2^ test was used for statistical analysis: χ.^2^ = 3.121, P = .037

### Factors potentially influencing the HHS score at the last follow-up

Multivariate linear regression analysis showed that different cup types, upward movement of the HCOR influenced the HHS score at the last follow-up (*p* < 0.001, *p* = 0.005, respectively), whereas age, gender, BMI, laterality, and the preoperative HHS score did not (Table 4).

**Table 4:** Multivariate linear regression of factors on the HHS score at the last follow-up

	Beta	*P* value	VIF
Age	0.022	0.806	1.122
Gender	0.454	0.065	8.028
BMI	0.026	0.778	1.217
Laterality	-0.429	0.083	8.199
the HHS score before surgery	0.071	0.451	1.228
3D printed cups vs non-3D printed cups	-0.603	< 0.001	2.427
The upward movement of HCOR at the last follow-up	-0.324	0.005	1.711

*HHS* Harris Hip Score, *VIF* Variance Inflation Factor, *BMI* Body mass index, *HCOR* Hip center of rotation

### Complications

None of the patients exhibited PJI, loosening, dislocation, deep venous thrombosis, or nerve palsy. One patient in the observation group and three in the control group had poor wound healing with oozing, which was resolved by timely dressing change and prolonged antibiotic use. In addition, one patient in the observation group and two in the control group had mild persistent pain, leading to anxiety, for 24 months postoperatively. This pain was not effectively treated with non-steroidal anti-inflammatory drugs.

## Discussion

How to improve the efficacy of revision THA has always been a hot-spot issue. Re-revision after failed revision surgery is not only an extremely complex operation with long treatment duration, many postoperative complications, and a high mortality rate, but also places great physiological, psychological, and economic burdens on the patient. A review of the literature confirmed that this is the first study to assess the new 3D-printed TT acetabular cup for use in revision THA. The results showed that this acetabular component is a reliable option for revision surgery, even in cases with severe acetabular bone loss.

Baauw et al. [13] reported that a cage is still the most frequently used technique in revision surgery. A cage that spans the ilium and ischia reconstructs the bone and provides initial stability, but does not support bone ingrowth or long-term biological fixation; in the long term, some cages eventually break down or loosen due to fatigue [14, 15]. There has been a steady increase in the use of porous titanium customized acetabular implants in revision THA. These implants provide an individualized fit for each patient and achieve satisfactory results, including improvement in hip joint function and good stability even with severe acetabular bone loss or pelvic discontinuity [16–18]. However, customized implants are not indispensable if bone loss is not severe.

An important feature of EBM is the realization of off-the-shelf 3D-printed TT acetabular cups. These cups have the advantages of decreased economic burden and reduced preoperative preparation duration compared to customized implants. The 3D-printed TT acetabular cups can be used alone for mild bone loss, or in combination with other surgical approaches for severe bone loss. Satisfactory clinical and radiographic outcomes of off-the-shelf 3D-printed TT cups in primary THA have also encouraged their use in revision surgeries. The DELTA-TT cup (Lima Corp., Udine, Italy) is manufactured by EBM and has a highly porous, hemispherical, multi-hole design, similar to Aikang TT cups. Geng et al. [19] reported excellent results in a cohort of 92 patients who underwent primary THA using DELTA-TT cups after a mean follow-up duration of 48.2 months. The survival rate of the cups was 100%, with no signs of cup loosening. Perticarini et al. [20] studied 133 cases of primary or revision THA using DELTA-TT cups and reported similar results, i.e., a 99.5% survival rate and excellent functional scores after a minimum of 5 years of follow-up. They suggested that this implant is optimal for primary surgery in cases of severe acetabular disease.

In the present study, although both cup types effectively alleviated pain, the 3D-printed cup was associated with higher HHS and SF-36 scores. A similar outcome was reported by Wan et al. [21] in a small series of 22 patients with 3D-printed cups and 20 patients with non-3D-printed cups. They reported that patients in the 3D-printed group achieved better functional scores, including HHS and SF-36, at 3, 6, and 12 months postoperatively. This difference was closely related to the HCOR and LLD [22]. One of main aims of surgery is to restore the normal HCOR and LLD. Excessive HCOR affects the stress distribution in soft tissues around the hip, causing decreased strength of the gluteus medius and adductor muscles, resulting in hip joint dysfunction, dislocation, and accelerated wear of the polyethylene liner, which has a significant impact on the early and long-term survival of the prosthesis [22]. Better postoperative recovery in our 3D-printed group was confirmed on the basis of upward movement of the HCOR in radiographs. The multivariate linear regression analysis also confirmed that upward movement of the HCOR influenced the HHS score at the last follow-up. The reason for the difference in HCOR between the two groups may be related to the diversity of the augment types in the observation group. Usually, in order to reduce the area of bone defect and increase the contact area between the cup and the acetabulum, the surgeon would grind the acetabulum appropriately deeper, which would cause the upward of the HCOR. Since the different shaped augments in the observation group can match the bone defect, the surgeon can allay these concerns and restored the normal HOCR as much as possible.Initial stability and secondary bone growth of cups were also evaluated in this study. Although loosening did not occur with either 3D-printed or conventional cups, a significant difference was observed in the rate of bone ingrowth between the two groups (91.30 and 70.59%, respectively) due to the highly porous surface and higher friction coefficient of the 3D-printed cups. TT, which consists of interconnected cells that form multi-planar hexagons, is a highly porous honeycomb structure that can mimic the trabecular morphology of human bone [23]. Many *in intro* studies reported that TT produced by EBM has good osteoinductive properties that stimulate vascularization, and osteoblast proliferation and differentiation [24, 25]. A sheep model study conducted by Declan et al. [26] demonstrated that, compared to traditional porous coatings, TT provided an optimized microenvironment for bone growth.

Melancon et al. [27] reported that the porosity and pore size of TT were key factors affecting bone growth, by affecting the transmission of fluids and providing a favorable microenvironment for the attachment and proliferation of osteoblasts. Additionally, the rough morphology of the pores increased the surface area for cell attachment and improved the surface energy of the material. Human trabecular bone has a porosity of 50–90% and pore size of 1,000 μm, and is an interconnected and open-porous structure. A porosity of 60–80% and pore size of 300–1,200 μm are suitable for cell growth and proliferation [28]. Porosity and pore size can be freely regulated and controlled using EBM during the additive manufacturing process. Three previous studies reported excellent stability and bone growth of the DELTA-TT cup, with an average porosity of 65% and mean pore size of 640 μm. De Meo et al. [29] observed no RLLs or signs of migration in 52 patients who underwent revision using Delta TT cups after a mean follow-up of 48.3 months, except in 6 patients who underwent re-revision. Relatively poorer outcomes were achieved by Gallart et al. [22] in 67 revision patients, and by Steno et al. [30] in 81 revision patients. In this study, an Aikang cup with an average porosity of 80% and pore size of 600–1,000 μm showed better bone growth, with no patients requiring re-revision for AL.

In addition to the aforementioned advantages, Dall'Ava et al. [31] revealed that the existence of titanium beads on three types of off-the-shelf 3D-printed TT cups was a by-product of the manufacturing process, where the beads may be released in the body. Bistolfi et al. [32] reported no difference in body titanium levels, as measured in blood and urine samples, between 3D-printed and conventional titanium cups. Research on this topic is limited. It is unclear whether titanium adversely affects clinical outcomes; more research is needed. Another problem is that the cost of 3D-printed cups is often higher than traditional cups due to the need for newer technologies in the manufacturing process. In China, a developing country, some families are really poor financially, but revision THA has to be done. Therefore, the cost of the prosthesis is a great concern for patients, which is one of the main reasons why 3D-printed cups are not widely used at present. However, we recommend the use of 3D printed TT acetabular cups for patients with severe osteoporosis and for young patients undergoing revision surgery due to its better bone growth. This study had several limitations. First, it was a retrospective, single center study. We plan to conduct a multicenter, prospective randomized controlled trial in the future to generate higher-level evidence. Another limitation was the inadequate follow-up duration. Long-term follow-up is indispensable for the assessment of clinical and radiological outcomes, complications, and cup survival rates.

The new off-the-shelf 3D-printed TT acetabular cup demonstrated encouraging short-to mid-term clinical outcomes in Chinese patients. It can effectively relieve pain, improve hip function, provide satisfactory biological fixation and high survival rate. But further follow up is necessary to assess its long-term outcomes.

## Data Availability

The final dataset will be available from the corresponding author.

## Abbreviations

THA: Total hip arthroplasty
AL: Aseptic loosening
3D: Three-dimensional
EBM: Electron beam melting
TT: Trabecular titanium
PJI: Periprosthetic joint infection
HCOR: Hip center of rotation
LLD: Limb-length discrepancy
RLL: Radiolucent line
